# Calcium-chelating alizarin and other anthraquinones inhibit biofilm formation and the hemolytic activity of *Staphylococcus aureus*

**DOI:** 10.1038/srep19267

**Published:** 2016-01-14

**Authors:** Jin-Hyung Lee, Yong-Guy Kim, Shi Yong Ryu, Jintae Lee

**Affiliations:** 1 School of Chemical Engineering, Yeungnam University, Gyeongsan, 712-749, Republic of Korea; 2 Korea Research Institute of Chemical Technology, Daejeon, 305-606, Republic of Korea

## Abstract

Staphylococcal biofilms are problematic and play a critical role in the persistence of chronic infections because of their abilities to tolerate antimicrobial agents. Thus, the inhibitions of biofilm formation and/or toxin production are viewed as alternative means of controlling *Staphylococcus aureus* infections. Here, the antibiofilm activities of 560 purified phytochemicals were examined. Alizarin at 10 μg/ml was found to efficiently inhibit biofilm formation by three *S. aureus* strains and a *Staphylococcus epidermidis* strain. In addition, two other anthraquinones purpurin and quinalizarin were found to have antibiofilm activity. Binding of Ca^2+^ by alizarin decreased *S. aureus* biofilm formation and a calcium-specific chelating agent suppressed the effect of calcium. These three anthraquinones also markedly inhibited the hemolytic activity of *S. aureus*, and in-line with their antibiofilm activities, increased cell aggregation. A chemical structure-activity relationship study revealed that two hydroxyl units at the C-1 and C-2 positions of anthraquinone play important roles in antibiofilm and anti-hemolytic activities. Transcriptional analyses showed that alizarin repressed the α-hemolysin *hla* gene, biofilm-related genes (*psmα*, *rbf*, and *spa*), and modulated the expressions of *cid*/*lrg* genes (the holin/antiholin system). These findings suggest anthraquinones, especially alizarin, are potentially useful for controlling biofilm formation and the virulence of *S. aureus*.

Most bacteria are likely to form biofilms that attach to living or abiotic surfaces using self-produced extracellular polymeric substances, and thus, biofilms are ubiquitous in natural, medical, and engineering environments^1^. Biofilms exhibit reduced sensitivity to conventional antimicrobial agents, host defenses, and external stresses, and thus, contribute to the bacterial persistence in chronic infections^2^,^3^. Since biofilm formation is a mechanism of antibiotic resistance, it is important to identify novel compounds capable of inhibiting biofilms without allowing bacteria to develop drug resistance.

Biofilm formation by *Staphylococcus aureus* and *Staphylococcus epidermidis* is of particular concern in the medical field, and *S. aureus* has caused numerous outbreaks of nosocomial infections^4^. Furthermore, the emergence of multidrug-resistant strains, such as, methicillin-resistant *S. aureus* (MRSA) and vancomycin-methicillin-resistant *S. aureus* has become a serious threat. These bacteria can secrete exotoxins, such as, hemolysin, enterotoxins, coagulase, TSST-1, and protein A, which are associated with specific diseases^5^, and can form biofilms on a variety of surfaces, including those of catheters, implants, prosthetics, and medical equipment^2^. Diverse mechanisms and environmental cues, for example, quorum sensing, c-di-GMP, protease, DNase, cis-2-decenoic acid, d-amino acids, phenol-soluble polypeptides, and pH, contribute to biofilm formation by *S. aureus*^6^,^7^. In addition, *S. aureus* produces α-toxin, which causes hemolysis and contributes to biofilm formation^8^. Hence, we sought to understand how biofilm inhibitors control biofilm formation by *S. aureus*.

Plant secondary metabolites are major sources of antimicrobial agents and other pharmaceuticals^9^, and several plant-derived biofilm inhibitors have been identified and shown to possess antibiofilm activity against *S. aureus*, examples include; magnolol^10^, ellagic acid^11^, tannic acid^12^, quercetin^13^, ginkgolic acids^14^, eugenol^15^, and flavonoids^16^. It has also been reported staphylococcal biofilm formation is inhibited by several plant essential oils^17^,^18^,^19^,^20^,^21^,^22^. However, the identification of active compounds in plant extracts and essential oils often requires extensive investigation to identify active components, and thus, only a limited number of organic biofilm inhibitors have been identified.

The goal of this work was to identify novel antibiofilm compounds against *Staphylococcus* species (including MRSA) from among 560 purified phytochemicals. Structure-activity analysis, confocal microscopy, slime analysis, hemolysis analysis, a cell aggregation assay, and transcriptional analysis were used to elucidate the mechanisms responsible for the inhibition of biofilm formation and toxin production.

## Results

### Alizarin inhibited biofilm formation by *S. aureus* and *S. epidermidis* without affecting planktonic cell growth

**Figure 1:**
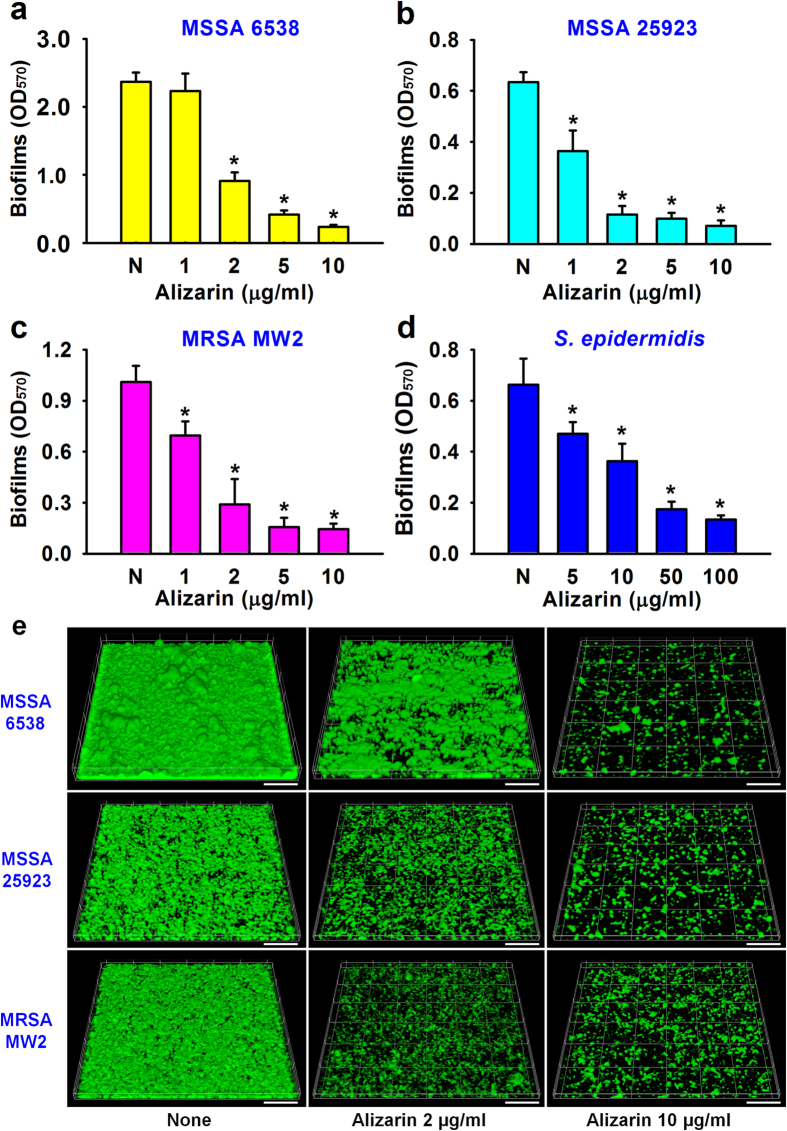
Antibiofilm activities of alizarin against *S. aureus* and *S. epidermidis*. The antibiofilm activities (OD_570_) of alizarin were determined against two methicillin-sensitive *S. aureus* strains (MSSA, ATCC 25923 and ATCC 6538), a methicillin-resistant *S. aureus* strain (MRSA, MW2) (**a–c**), and *S. epidermidis* (ATCC 14990) (**d**). Two independent experiments were conducted (12 wells per sample); error bars indicate standard deviations. *P < 0.05 versus non-treated controls (N or None). Biofilm formation on glass was observed by confocal laser microscopy (**e**). Scale bars represent 50 μm.

Screening of the 560 phytochemicals for antibiofilm activity against *S. aureus* MSSA 6538 on 96-well polystyrene plates showed that alizarin at 10 μg/ml most inhibited *S. aureus* biofilm formation. Twenty of the 560 chemicals inhibited *S. aureus* biofilm formation by >60% and nine enhanced biofilm formation by >60% (Supplementary Fig. S1). Further experiments showed that the addition of alizarin (0, 1, 2, 5, 10, 50, or 100 μg/ml) at the beginning of bacterial culture dose-dependently inhibited biofilm formation by all three *S. aureus* strains (MSSA 6538, MSSA 25923, and MRSA MW2) and a *S. epidermidis* strain (ATCC 14990) (Fig. 1a–d). Specifically, alizarin (at 10 μg/ml) decreased biofilm formation by all three *S. aureus* strains by ≥90%, whereas in the case of *S. epidermidis*, 50 μg/ml was required to inhibit biofilm formation by ≥70%. Unlike Gram-positive bacteria, biofilm formation by two Gram-negative bacteria (*Escherichia coli* O157:H7 and *Pseudomonas aeruginosa* PAO1) was unaffected by alizarin at concentrations up to 100 μg/ml (Supplementary Fig. S2).

Confocal laser microscopy was used to analyze changes in biofilm formation on glass, and in-line with biofilm data obtained using 96-well polystyrene plates (Fig. 1a–c), fluorescent images indicated alizarin (0, 2, or 10 μg/ml) dose-dependently inhibited *S. aureus* biofilm formation (Fig. 1e). Biofilm inhibition was further confirmed by COMSTAT biofilm analysis, which showed alizarin (at 10 μg/ml) reduced all three measured biofilm parameters (biomass, mean thickness, and substratum coverage) of the three *S. aureus* strains by ≥80% versus untreated controls (Supplementary Table S1). For example, *S. aureus* MSSA 6538 biofilm biomass was reduced from 12 μm^3 ^μm^−2^ to 0.9 μm^3 ^μm^−2^ in the presence of alizarin at 10 μg/ml.

Counts of viable biofilm cells were performed to confirm biofilm inhibition by alizarin. In agreement with the results of other biofilm assays, alizarin dose-dependently reduced viable cell numbers in the biofilms of the four *Staphylococcus* strains. For example, alizarin at 10 μg/ml reduced the number of viable cells in MSSA 6538 and MRSA MW2 biofilms by more than 7-fold versus untreated controls (Supplementary Table S2).

**Figure 2:**
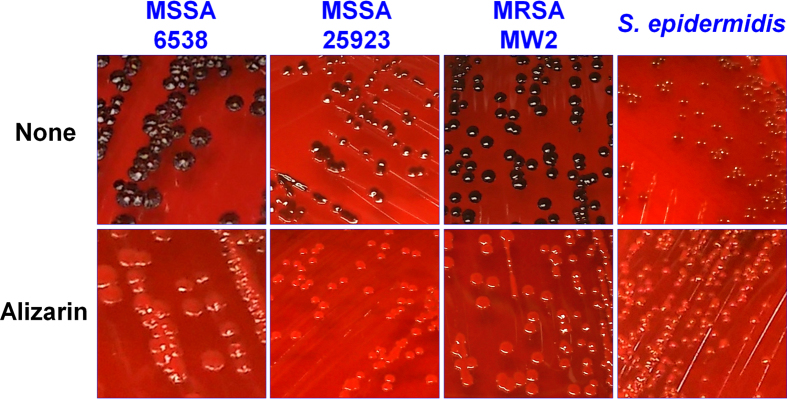
Inhibition of slime production by alizarin. Slime production was analyzed using Congo red agar plates. Three *S. aureus* strains (MSSA 25923, MSSA 6538, and MRSA) and a *S. epidermidis* strain were cultured with and without alizarin (20 μg/ml) on Congo red agar plates for 24 h at 37 °C. Four independent experiments were conducted and one set of representative results is shown. None represents non-treated controls.

Slime detection using Congo red plates is conventionally used to detect biofilm-forming staphylococci^23^, and consistent with the 96-well plate and microscopic results, slime production by all four staphylococci strains was markedly reduced by alizarin at 20 μg/ml (Fig. 2). Noticeably, *S. epidermidis* produced least slime, whereas the two *S. aureus* strains (MSSA 6538 and MRSA MW2) produced large amounts.

The antimicrobial activity of alizarin was investigated by measuring minimum inhibitory concentration (MICs), and the MICs of alizarin against *S. aureus* MSSA 6538 and *S. epidermidis* were found to be >1000 μg/ml, which were consistent with previously reported values^24^. Notably, its MIC against *S. aureus* was 100-times higher than the concentration (10 μg/ml) required for antibiofilm activity. Furthermore, alizarin at concentrations up to 20 μg/ml did not retard the growth of *S. aureus* planktonic cells, although at 200 μg/ml it had a slight inhibitory effect (Supplementary Fig. S3). These findings show the reduced biofilm formation caused by alizarin was due to its antibiofilm activity and not to its antimicrobial activity.

### Antibiofilm activities of anthraquinone derivatives against *S. aureus*

**Figure 3:**
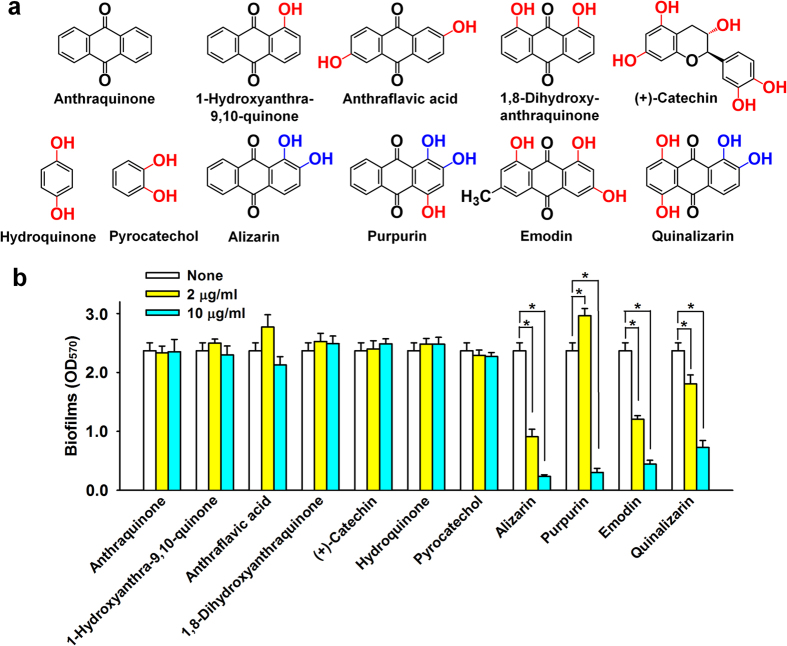
Inhibition of biofilm formation by alizarin-related chemicals. Chemical structures are shown (**a**). Hydroxyl groups are shown in red and the two hydroxyls at C-1 and C-2 of anthraquinone are shown in blue. Biofilm formation by MSSA 6538 was quantified in the presence of selected chemicals after incubation for 24 h in 96-well polystyrene plates without shaking (**b**). At least two independent experiments were conducted (6 wells per sample). Error bars indicate standard deviations. *P < 0.05 versus non-treated controls (None).

Since alizarin (1,2-dihydroxyanthraquinone) is an anthraquinone, we also investigated the antibiofilm activities of eleven other anthraquinone-related compounds (Fig. 3a). It was found alizarin, emodin, purpurin, and quinalizarin at 10 μg/ml markedly inhibited *S. aureus* MSSA 6538 biofilm formation by ≥70% versus untreated controls, whereas the other seven compounds had no significant effect (Fig. 3b).

Interestingly, antibiofilm activity against *S. aureus* was found to be closely related to the number and position of hydroxyl units (Fig. 3). Hydroxyls at the C-1 and C-2 positions of the anthraquinone skeleton appeared to be important for antibiofilm activity, because alizarin, purpurin, and quinalizarin possess a hydroxyl group at both positions (Fig. 3a). However, pyrocatechol (1,2-dihydroxybenzene), which has two hydroxyl units in a benzene structure lacked inhibitory activity, indicating that the anthraquinone backbone and the C-1 and C-2 hydroxyl units are required for antibiofilm activity. Emodin, which has a methyl group at the C-6 position, inhibited planktonic growth, as previously reported^25^, and the additional hydroxyl units at positions other than C-1 and C-2 of purpurin and quinalizarin had minor effects on antibiofilm activity. The same pattern of antibiofilm activities was observed for the other two *S. aureus* strains, MSSA 25923 and MRSA MW2 (Supplementary Fig. S4). Because alizarin reduced biofilm formation most, we focused on alizarin for further study.

### The inhibitory activity of alizarin and the effect of Ca^2+^

**Figure 4:**
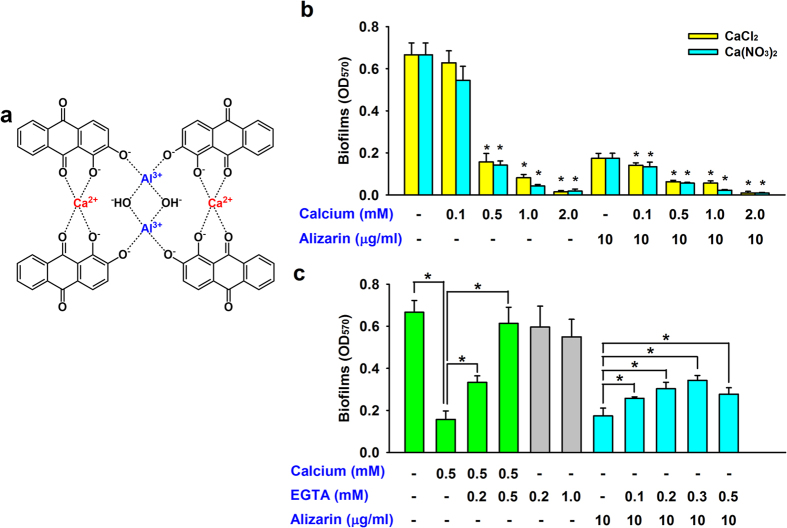
The effect of Ca^2+^ and alizarin on biofilm formation by *S. aureus*. Structures of the alizarin/calcium/aluminum complex (**a**)^26^. Biofilm formation by MSSA 6538 was quantified in M9 minimal medium supplemented with Ca^2+^ (**b**). EGTA (a calcium-specific chelator) complements the effect of calcium and decreases the effect of alizarin (**c**). At least two independent experiments were conducted (6 wells for each sample). Error bars indicate standard deviations. *P < 0.05 for the indicated pairs of groups.

Alizarin forms a calcium/aluminum complex (Fig. 4a)^26^, and it has been previously shown that Ca^2+^ participates in^27^ and inhibits *S. aureus* biofilm formation^28^. When we investigated the effect of calcium and alizarin on *S. aureus* MSSA 6538 biofilm formation, two sources of Ca^2+^ (CaCl_2_ and Ca(NO_3_)_2_) were found to dose-dependently and similarly inhibit *S. aureus* biofilm formation (Fig. 4b). In addition, the antibiofilm activity of alizarin was augmented by Ca^2+^ (Fig. 4b). Furthermore, the inhibitory effect of Ca^2+^ disappeared in the presence of EGTA (ethylene glycol tetraacetic acid; a calcium-specific chelating agent), whereas EGTA alone at concentrations up to 1 mM did not influence biofilm formation (Fig. 4c). Furthermore, the addition of EGTA in the presence of alizarin partially decreased the antibiofilm effect of alizarin (Fig. 4c). These results suggest alizarin inhibits *S. aureus* biofilm formation with the involvement of Ca^2+^.

### Inhibition of the hemolytic effects of *S. aureus* by alizarin and other anthraquinones

**Figure 5:**
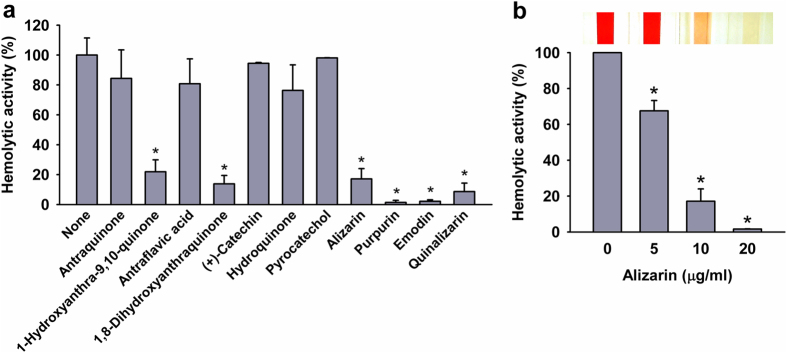
Anti-hemolytic activities of alizarin and other anthraquinones. The hemolysis of human blood by *S. aureus* MSSA 6538 was quantified in the presence of anthraquinone-related compounds at 10 μg/ml (**a**) or alizarin (0, 5, 10, or 20 μg/ml) (**b**) after incubation for 20 h. Pictures of spectrophotometer cuvettes are shown. At least two independent experiments were conducted. *P < 0.05 versus non-treated controls (None or 0).

*S. aureus* produces α-toxin that causes hemolysis^29^ and contributes to biofilm formation^8^, and thus, we investigated the effects of alizarin and of 10 other anthraquinone-related compounds on blood hemolysis by *S. aureus* (Fig. 5). In accord with observed antibiofilm activities, alizarin, emodin, purpurin, and quinalizarin at 10 μg/ml inhibited the hemolytic activity of *S. aureus* MSSA 6538 by ≥70% versus untreated controls (Fig. 5a). 1-Hydroxyanthra-9,10-quinone and 1,8-dihydroxyanhraquinone also showed anti-hemolytic activity, indicating that the hydroxyl unit at C-1 plays an important role in the inhibition of hemolysis by *S. aureus*. This result also suggests that biofilm inhibitions by alizarin, purpurin, and quinalizarin are associated with the inhibition of hemolytic activity. Furthermore, alizarin (0, 5, 10, or 20 μg/ml) dose-dependently reduced hemolysis by *S. aureus*, and at 20 μg/ml (84 μM) alizarin completely abolished the hemolytic activity of *S. aureus* (Fig. 5b).

### Alizarin, purpurin, and quinalizarin increased cell aggregation

**Figure 6:**
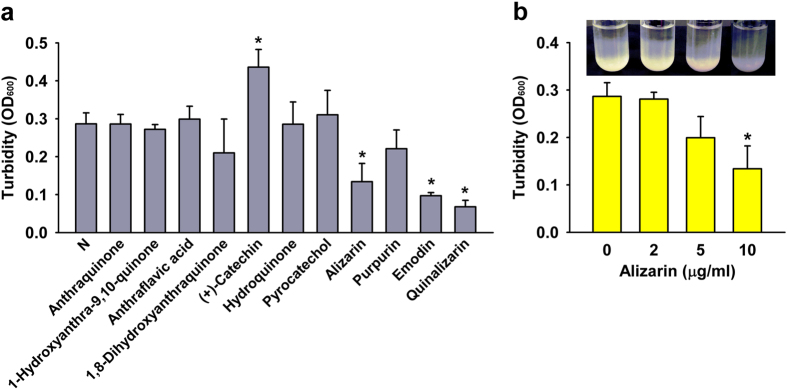
Effects of alizarin-related compounds on cell aggregation. *S. aureus* MSSA 6538 cells were grown for 20 h in the presence of alizarin-related compounds (10 μg/ml) (**a**) or in the presence of alizarin (0, 2, 5, or 10 μg/ml) (**b**). Absorbances of the top 1 ml portions of test tubes were measured at OD_600_. Tested tubes are shown. At least two independent experiments were conducted. *P < 0.05 versus non-treated controls (N or 0).

Since polyphenols bind to proteins and cause the formation of insoluble aggregates^30^, we investigated the abilities of the 11 anthraquinone-related compounds to induce aggregation. Interestingly, alizarin and quinalizarin caused obvious aggregation of *S. aureus* MSSA 6538, while the other compounds did not (Fig. 6a). In the case of emodin, low optical density was due to low cell growth, but not due to cell aggregation. On the other hand, alizarin dose-dependently increased cell aggregation (Fig. 6b). Furthermore, aggregation results were generally in-line with the observed antibiofilm and anti-hemolytic activities of alizarin and quinalizarin.

### Alizarin modulated the expressions of biofilm- and toxin-related genes

**Figure 7:**
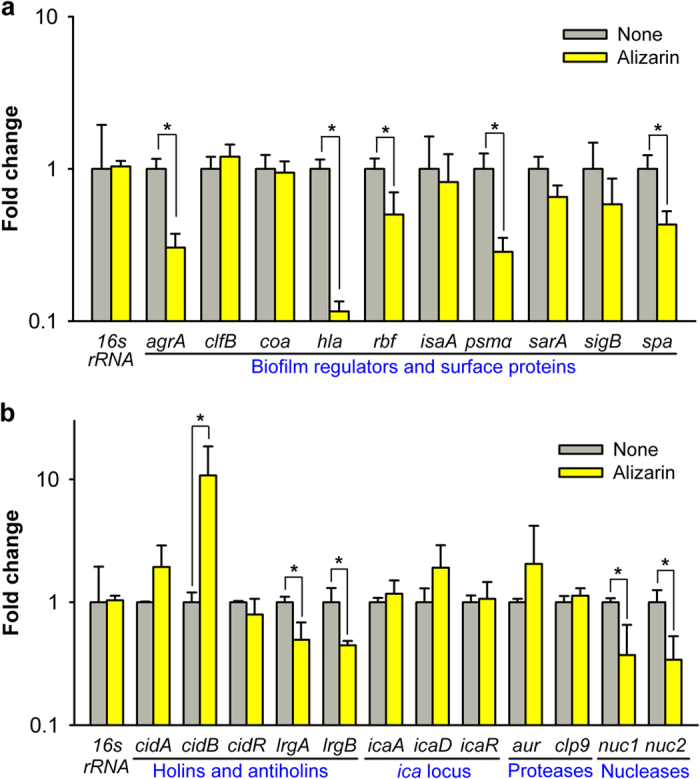
Transcriptional profiles of *S. aureus* cells treated with or without alizarin. *S. aureus* MSSA 6538 was cultivated to an A_600_ of 1 and then incubated with or without alizarin (20 μg/ml) for 2 h with shaking at 250 rpm. Transcriptional profiles were measured by qRT-PCR. The expression level of 16s rRNA was used to normalize the expressions of genes of interest. Fold changes represent changes in transcriptions of treated versus untreated *S. aureus*. The experiment was performed in triplicate (6 qRT-PCR reactions were performed per gene). *P < 0.05 versus non-treated controls (None).

To investigate the molecular mechanism underlying the antibiofilm and anti-hemolytic activities of alizarin in *S. aureus* MSSA 6538, we examined the differential expressions of 22 biofilm- and toxin-related genes using planktonic *S. aureus* cells by real-time qRT-PCR. As shown in Fig. 7, alizarin altered the expressions of many genes. Of particular note, and in accord with its observed inhibition of hemolytic activity (Fig. 5), alizarin repressed expression of the α-hemolysin gene (*hla*) by 9-fold (Fig. 7a), and significantly repressed the expressions of the biofilm-related genes, *psmα* (phenol soluble modulins α), *rbf* (clumping factor B), and *spa* (surface protein A) (Fig. 7a). In addition, alizarin altered the expressions of the *cid* and *lrg* genes, which constitute the holin-antiholin system. Notably, alizarin induced *cidB*, which encodes for a holin-like protein, by more than 13-fold, but repressed *lrgAB*, which encode for antiholin proteins. However, the expressions of other biofilm-related genes, such as, intercellular adhesion locus genes (*icaA, icaD*, and *icaR*), proteases genes (*aur* and *clp9*), and other biofilm regulators (*clfB*, *coa isaA*, and *sarA*) were relatively unaffected by alizarin. In addition, alizarin repressed the expressions of *agrA* and of the nuclease genes (*nuc1* and *nuc2*) (Fig. 7). Although it has been established that *agrA* quorum-sensing causes dispersal^31^, alizarin did not induce biofilm dispersal (data not shown), which suggests the mode of action of alizarin is less associated with biofilm dispersal systems, such as, *agr* quorum sensing or the actions of proteases and nucleases.

## Discussion

The present study demonstrates for the first time that alizarin, purpurin, and quinalizarin exhibit antibiofilm and anti-hemolytic activity against *S. aureus*. In addition, it identifies chemical structure-activity relationships and partially reveals the action mechanisms underlying their antibiofilm effects.

Hydroxyanthraquinones are found in various plants, and mixtures of anthraquinones have long been employed in medical preparations as laxatives^32^. Furthermore, the toxicities of alizarin, purpurin, and quinalizarin are relatively low as compared with many phenolic agents^32^. Alizarin (also known as Turkey Red) was originally derived from the roots of the madder genus and has been used as a red dye. Alizarin stains ionic calcium in bones and for this reason has been widely used in studies on bone growth^26^. In the present study, alizarin at 10 μg/ml (100-times lower than its MIC) significantly inhibited biofilm formation by and the hemolytic activity of *S. aureus*, indicating that it is a non-toxic biofilm inhibitor.

One of objectives of the present study was to identify the structural motif present in anthraquinones responsible for antibiofilm and anti-hemolytic activities against *S. aureus*. Our results suggest that the anthraquinone backbone and two hydroxyl units at the C-1 and C-2 positions of the anthraquinone skeleton are required for the observed antibiofilm (Fig. 3) and anti-hemolytic effects of anthraquinones on *S. aureus* (Fig. 5). Many alizarin derivatives can be synthesized, and thus, further investigations of alizarin derivatives are probably worthwhile. Previously, it was reported that emodin reduced biofilm formation by another Gram-positive oral bacterium, *Streptococcus mutans*^33^. In the present study, the antimicrobial activity of emodin was found to be responsible for inhibiting biofilm formation by and the hemolytic activity of *S. aureus* (Figs 3 and 5).

Staphylococcal biofilms are encased in an extracellular matrix composed of proteins, polysaccharides, and extracellular DNA. The mechanism of biofilm formation by *S. aureus* is complicated and involves environmental factors, quorum sensing, proteases, DNase, several surface proteins, and other global regulators^6^,^7^. In the present study, we investigated the transcriptional levels of various biofilm- and toxin-related genes and found positive biofilm regulators (*psmα*, *rbf*, and *spa*) were repressed by alizarin (Fig. 7), which supports its biofilm reducing effect on *S. aureus*. Phenol-soluble modulins (PSMs) are a novel family of toxins and play multiple roles in the pathogeneses of staphylococcal infections, which typically involve blood cell lysis and biofilm development^34^,^35^. On the other hand, Rbf is an activator of biofilm formation by *S. aureus*^36^, and was found to promote virulence in a murine model of infection^37^. Furthermore, surface protein A (SpA) production has been reported to be essential for biofilm formation by *S. aureus*^38^. Thus, our findings show that alizarin down-regulates several important biofilm regulators in this bacterium.

*S. aureus* produces four hemolysins (alpha, beta, gamma, and delta), which have hemolytic, cytotoxic, and dermonecrotic properties^39^. In particular, α-toxin (Hla) causes hemolysis^29^ and contributes to biofilm formation^8^. In the present study, alizarin, purpurin, and quinalizarin showed antibiofilm and anti-hemolytic activities (Figs 3 and 5), and previous studies have shown that several flavonoids^16^, nerolidol^22^, stilbenoids^40^, and thermoresponsive oligo (*N*-vinylcaprolactam)^41^ have antibiofilm activity and anti-hemolytic activity against *S. aureus*. Thus, it appears there is a positive relation between antibiofilm and anti-hemolytic activities.

In the present study, alizarin markedly up-regulated the gene expression of holin-like protein (CidB) and down-regulated those of antiholin proteins (LrgAB) (Fig. 7). Holin (CidA) and antiholin (LrgA) may serve as molecular control elements of bacterial cell lysis and play significant roles during biofilm development^42^. Like *cidA* and *lrgA*, the *cidB* and *lrgB* genes encode homologous hydrophobic proteins, but the functions of these have not been well established^43^. Although the mechanism responsible for modulation of the Cid/Lrg system by alizarin is unclear, alizarin could affect bacterial cell wall integrity. Interestingly, our qRT-PCR data revealed a gene modulation pattern similar to that of a synthetic antibiofilm agent CCG-203592, which temporally down-regulates the expressions of *hla, lrgA, psmα*, and *spa*, but temporally up-regulates *cidA* in *S. aureus*^44^. Further genetic studies should provide more detail of the molecular mechanisms responsible for the effects of alizarin and its derivatives.

It is generally believed that cell aggregation is a prerequisite of biofilm development^7^. However in the present study, alizarin and quinalizarin increased cell aggregation (Fig. 6) but decreased biofilm formation (Fig. 3). Nevertheless, these results are in-line with those of a recent study on the effects of proanthoyanides on biofilm formation by *S. epidermidis*, in which it was proposed that inhibition of bacterial attachment is based on electrostatic repulsion and changes in hydrophobicity^45^.

Ca^2+^ plays a role in *S. aureus* biofilm formation^27^ and at millimolar concentrations has an inhibitory effect^28^. Furthermore, calcium addition has been reported to decrease α-hemolysin-induced hemolytic activity by *S. aureus*^46^. Our results suggest that alizarin and complexed Ca^2+^ at micromolar concentrations effectively inhibit the biofilm and hemolytic activities of *S. aureus* (Figs 3 and 5). However, it remains to be determined how alizarin and Ca^2+^ function at the molecular level in *S. aureus* cells.

Because the long-term use of antibiotics has generated multidrug resistant bacteria like MRSA, novel strategies are urgently required to control antibiotic resistant *Staphylococcus* strains, and strategies based on inhibiting biofilm formation and toxin production offer an alternative means of reducing bacterial virulence. The present study shows for the first time that the alizarin exhibits antibiofilm and anti-hemolytic activities and down-regulates the expressions of various biofilm- and toxin-related genes, and thus, identifies alizarin and its derivatives as potential antivirulence compounds against recalcitrant *S. aureus*.

## Methods

### Ethics statement

Hemolysis experiment was approved by the Ethical Committee of Yeungnam University, Gyeongsan, Korea and the methods were carried out as per the guidelines of the Ethical Committee of Yeungnam University. All participants provided written informed consent for blood collection and research.

### Bacterial strains, growth measurements, and materials

The following bacterial strains were used in the present study; methicillin-sensitive *S. aureus* strains (MSSA; ATCC 25923 and ATCC 6538), a methicillin-resistant *S. aureus* strain (ATCC BAA-1707, MW2), *S. epidermidis* (ATCC 14990), *Pseudomonas aeruginosa* PAO1 (ATCC 15692), and *Escherichia coli* O157:H7 (ATCC 43895, EDL933). Experiments were conducted on the two MSSA strains and *S. epidermidis*, *P. aeruginosa* PAO1, and *E. coli* O157:H7 at 37 °C in LB medium, and on the MRSA strain in LB medium containing 0.2% glucose. For cell growth measurements, colony forming units (CFUs) were measured by spreading cell cultures on LB agar plates. For the MIC experiment, cells were inoculated with overnight culture at a dilution of 1:100 in LB medium and cultured for 24 h at 37 °C. After serial dilutions, cultures were spread on LB agar plates, incubated for 24 h at 37 °C, and cell colonies were counted. Each experiment was performed using at least two independent cultures.

We have established a library of 560 phytochemicals, and deposited it in the Natural Product Library in the Korea Chemical Bank (http://www.chembank.org, Daejeon, Republic of Korea). These 560 compounds were purified from various plant sources and included terpenoids, flavonoids, polyphenols, and saponins, as we previously described^47^. All were dissolved in dimethyl sulfoxide (DMSO). Alizarin and ten other anthraquinone-related compounds, namely, anthraflavic acid, anthraquinone, (+)-catechin, 1,8-dihydroxyanthraquinone, emodin, 1-hydroxyanthra-9,10-quinone, hydroquinone, purpurin, pyrocatechol, and quinalizarin were purchased from Sigma-Aldrich (St. Louis, USA).

### Crystal-violet biofilm assay

A static biofilm formation assay was performed on six bacterial strains (MSSA 6538, MSSA 25923, MRSA MW2, *S. epidermidis*, *Pseudomonas aeruginosa* PAO1, and *Escherichia coli* O157:H7) in 96-well polystyrene plates (SPL Life Sciences, Korea), as previously reported^48^. Briefly, cells were inoculated into LB medium (total volume 300 μl) at an initial turbidity of 0.05 at 600 nm. Antibiofilm agents were added at different concentrations at inoculation and cultured for 24 h without shaking at 37 °C. To quantify biofilm formation, biofilms were stained with crystal violet for 20 min, dissolved in 300 μl of 95% ethanol, and absorbances were measured at 570 nm (OD_570_). Cell growths in 96-well plates were also measured at 620 nm (OD_620_). Biofilm formation and static cell growth results are presented as the averages of two independent cultures of twelve replicate wells.

### Biofilm cell counting assay

To confirm biofilm inhibition, we performed viable counts on biofilm cells. Biofilm cells were formed in 96-well polystyrene plates for 24 h with or without alizarin (as mentioned above), and the biofilms obtained were washed three times with phosphate-buffered saline (PBS). Biofilms were then resuspended in 300 μl PBS, pipetted vigorously for 60 sec, vortexed for 30 sec (to disrupt the biofilms), serially diluted, and plated on LB agar plates. CFUs were counted after overnight incubation at 37 °C. To check complete biofilm disruption had been achieved, we used the crystal-violet biofilm assay after vigorous pipetting. Three independent experiments were conducted.

### Slime assay using Congo red agar (CRA)

Colony morphologies and phenotypic changes were investigated using CRA, as previously described^23^. The CRA was composed of 37 g/L of brain–heart infusion broth (BD Biosciences, Franklin Lakes, NJ, USA), 36 g/L of sucrose (Sigma, St. Louis, MO, USA), 15 g/L of agar (BD Biosciences, Franklin Lakes, NJ, USA), and 0.8 g/L of Congo red (Sigma, St. Louis, MO, USA). *Staphylococcus* cells (MSSA 6538, MSSA 25923, MRSA MW2, and *S. epidermidis*) on CRA were incubated with and without alizarin for 24 h at 37 °C before taking images. Four independent experiments were conducted.

### Confocal laser microscopy and COMSTAT analysis

Biofilm formations by *S. aureus* (MSSA 6538, MSSA 25923, and MRSA MW2) on glass were evaluated by confocal laser microscopy (Nikon Eclipse Ti, Tokyo) and compared with *S. aureus* biofilms grown in medium alone. *S. aureus* cells were stained with carboxyfluorescein diacetate succinimidyl ester (Catalog #:C34554 Invitrogen, Molecular Probes, Inc, Eugene, USA)^49^, which is a minimally fluorescent lipophile, but on entering cells esterases remove its acetyl groups to become markedly the fluorescent^50^. Hence, this fluorescent dye targets viable cells in biofilms. Briefly biofilms were allowed to form by incubating 96-well plates for 24 hr at 37 °C without shaking, washed with PBS twice, stained with carboxyfluorescein diacetate succinimidyl ester (2.8 μg/ml in PBS) for 20 min at 37 °C, and rewashed twice with PBS. Samples were visualized using a 40 x objective and an Ar laser (excitation 488 nm; emission 500 to 550 nm). Confocal images of same strains were captured using the same conditions. Color confocal images were constructed using NIS-Elements C version 3.2 (Nikon eclipse). At least 4 random positions in three independent cultures were subjected to analysis.

To quantify biofilm formation, color confocal images (20 image stacks) were converted to gray scale using ImageJ. COMSTAT biofilm software^51^ was used to determine biomasses (μm^3 ^per μm^2^), mean thicknesses (μm), and substratum coverages (%). Thresholding was fixed for all image stacks, and at least 4 positions and 20 planar images were analyzed per position.

### Hemolysis assay

Human red blood cell lysis efficacies were measured using whole cultures of *S. aureus* grown in the presence of biofilm inhibitors, as described previously^13^. Briefly, *S. aureus* cells (MSSA 6538) were diluted 1:100 in LB medium and cultured with or without test compounds for 20 h at 250 rpm. Cell cultures (cells and supernatants) were then added to the diluted human red blood cells (previously separated by centrifugation at 890 x g for 2 min and washed 3 times with PBS (330 μl red blood cells/10 ml of PBS buffer). To determine hemolytic activities, mixtures of blood and *S. aureus* (200 μl of cell culture) were incubated at 250 rpm for 1 h at 37 °C. Supernatants were collected by centrifugation at 16,600 x g for 10 min and optical densities were measured at 543 nm.

### Cell aggregation assay

Cell aggregation was analyzed as previously reported^52^. Briefly, *S. aureus* cells (MSSA 6538) were inoculated into 2 ml of LB medium in 14-ml test tubes with or without alizarin or alizarin-related compounds and incubated for 20 h with shaking at 250 rpm. Cell cultures (1 ml) were then collected by centrifugation at 16,600 x g for 2 min and cells were washed with PBS 3 times. Washed cells were resuspended in 3 ml of PBS in clean glass tubes and allowed to stand for 20 h at room temperature. Cell turbidities of the top portions of tubes were measured at OD_600_ using a spectrophotometer (UV/Vis, spectrophotometer, Optizen, Korea).

### RNA isolation

For qRT-PCR (quantitative real-time reverse transcription polymerase chain reaction) experiments, the RNAs of *S. aureus* cells were isolated using the following procedure. *S. aureus* cells (MSSA 6538) were inoculated into 25 ml of LB medium at 37 °C in 250 ml shake flasks with overnight cultures (1 : 100 dilution) and cultured for 3 h with shaking at 250 rpm. Alizarin was then added to a concentration of 20 μg/ml, at which it showed significant antibiofilm and anti-hemolytic activity, and incubated for 2 h. Before sample collection, RNase inhibitor (Ambion, TX, USA) was added and planktonic cells were immediately chilled for 30 sec with dry ice and 95% ethanol to prevent RNA degradation. Cells were then centrifuged at 16,600 x g for 1 min and the cell pellets obtained were immediately frozen with dry ice and stored at −80 °C. RNA was isolated using a Qiagen RNeasy mini Kit (Valencia, CA, USA). To remove all DNA, purified RNA was treated with 30 units of DNase I for 15 min. RNA quality was assessed using a NanoVue Plus (Biochrom Ltd., Cambridge, UK).

### qRT-PCR

qRT-PCR was used to assess the transcription levels of biofilm-related genes (*agrA, aur*, *cidA*, *cidB*, *cidR*, *clfB*, *clp9*, coa, *hla, icaA*, *icaD*, *icaR*, *isaA*, *lrgA*, *lrgB*, *nuc*1, *nuc*2, *psmα*, *rbf*, *sarA*, *sigB, and spa*) in *S. aureus* (MSSA 6538) cells. Gene specific primers were used for these genes and appropriate primers for 16s rRNA as a housekeeping control (Supplementary Table S3), which was used to normalize the expressions of genes of interest. The qRT-PCR method employed was adapted from a previous study^53^, and performed using a SYBR Green master mix (Applied Biosystems, Foster City, USA) and an ABI StepOne Real-Time PCR system (Applied Biosystems). Expression levels were determined using three independent cultures and six qRT-PCR reactions for each gene.

### Statistical analysis

Sample sizes of all experiments are indicated in ‘Methods’. Average values are expressed as means  ±  standard deviations, and the Student’s t-test was used to determine the significances differences between samples and non-treated controls. Statistical significance was accepted for p values < 0.05, and significant changes are indicated using asterisks in figures.

## Additional Information

**How to cite this article**: Lee, J.-H. *et al*. Calcium-chelating alizarin and other anthraquinones inhibit biofilm formation and the hemolytic activity of *Staphylococcus aureus*. *Sci. Rep*. **6**, 19267; doi: 10.1038/srep19267 (2016).

## Supplementary Material

Supplementary Information
