# Supplementary material for: Calcium-chelating alizarin and other anthraquinones inhibit biofilm formation and the hemolytic activity of Staphylococcus aureus
Source: Sci Rep. 2016 Jan 14;6:19267. doi: 10.1038/srep19267 (PMC4725881; doi:10.1038/srep19267)
Supplement: Supplementary Information [file srep19267-s1.pdf]

Supplementary Information

Calcium-chelating alizarin and anthraquinones inhibit biofilm formation and the hemolytic activity of *Staphylococcus aureus*

Jin-Hyung Lee[1]*, Yong-Guy Kim[1]*, Shi Yong Ryu[2], and Jintae Lee[1†]

[1]*School of Chemical Engineering, Yeungnam University, Gyeongsan, 712-749, Republic of Korea*

[2]*Korea Research Institute of Chemical Technology, Daejeon, 305-606, Republic of Korea*

Supplementary Figure S1. Histogram of *S. aureus* biofilm formation in the presence of each of the 560 phytochemicals. Biofilm screening for *S. aureus* MSSA 6538 was performed at a concentration of 10 μg/ml in 96-well plates after culture for 24 h. Numbers on the right indicate numbers of phytochemicals. Biofilm formation (%) on the Y-axis represents changes in biofilm formation, that is, biofilm formation in the presence of a phytochemical is expressed as a percentage of biofilm formation in its absence.

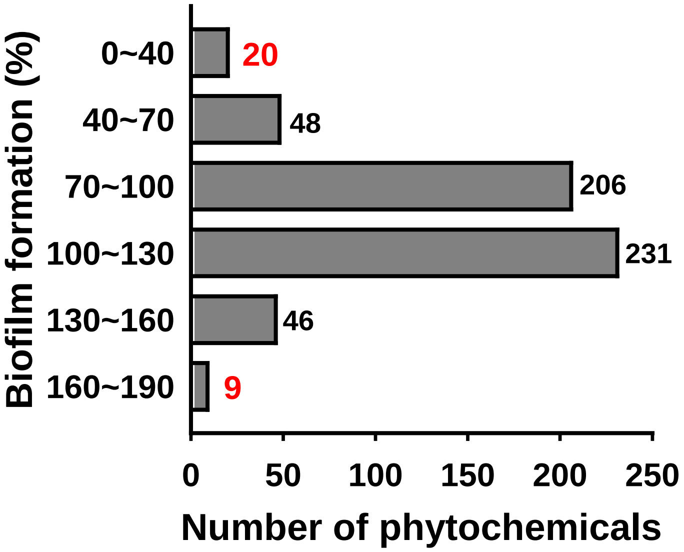

Supplementary Figure S2. Biofilm formation by *Escherichia coli* O157:H7 (a) or *Pseudomonas aeruginosa* PAO1 (b) in the presence of alizarin in LB medium after culture for 24 h. Two independent experiments were conducted (total 12 wells); error bars indicate standard deviations.

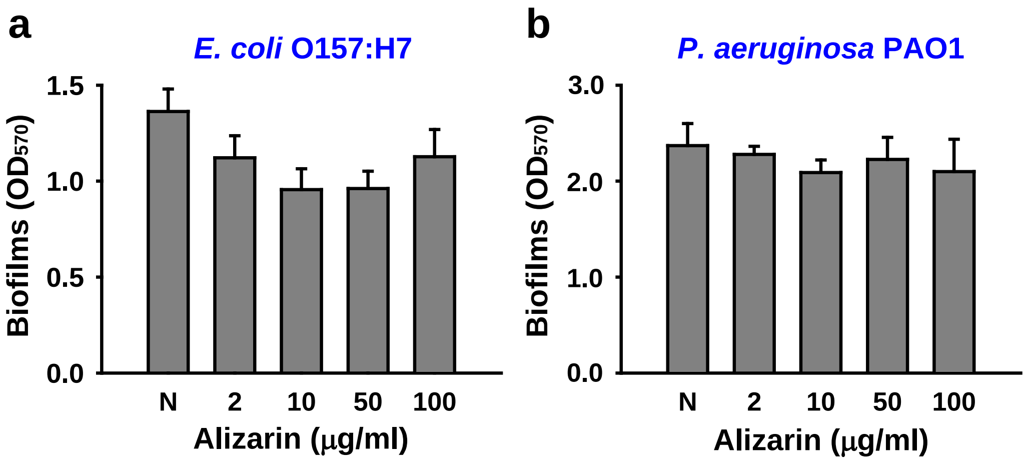

Supplementary Figure S3. Cell growth of *S. aureus* in the presence of alizarin. Planktonic cell growth of *S. aureus* MSSA 6538 was measured by counting CFUs in 250 ml-flasks agitated at 250 rpm.

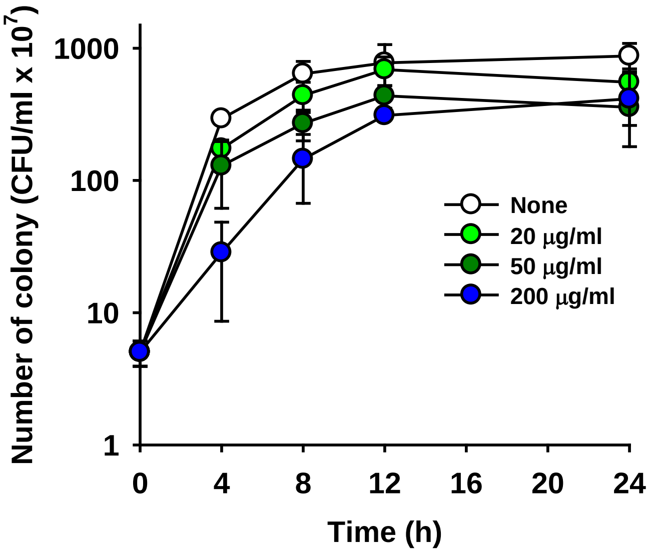

Supplementary Figure S4. Inhibitory effects of alizarin-related compounds on biofilm formation. Biofilm formations by MSSA 25923 (a) and MRSA MW2 (b) were quantified in the presence of the selected compounds at 0, 2, or 10 μg/ml after incubation for 24 h in 96-well plates without shaking. At least two independent experiments were conducted (total 12 wells). Error bars indicate standard deviations. *, *P*<0.05 versus non-treated controls.

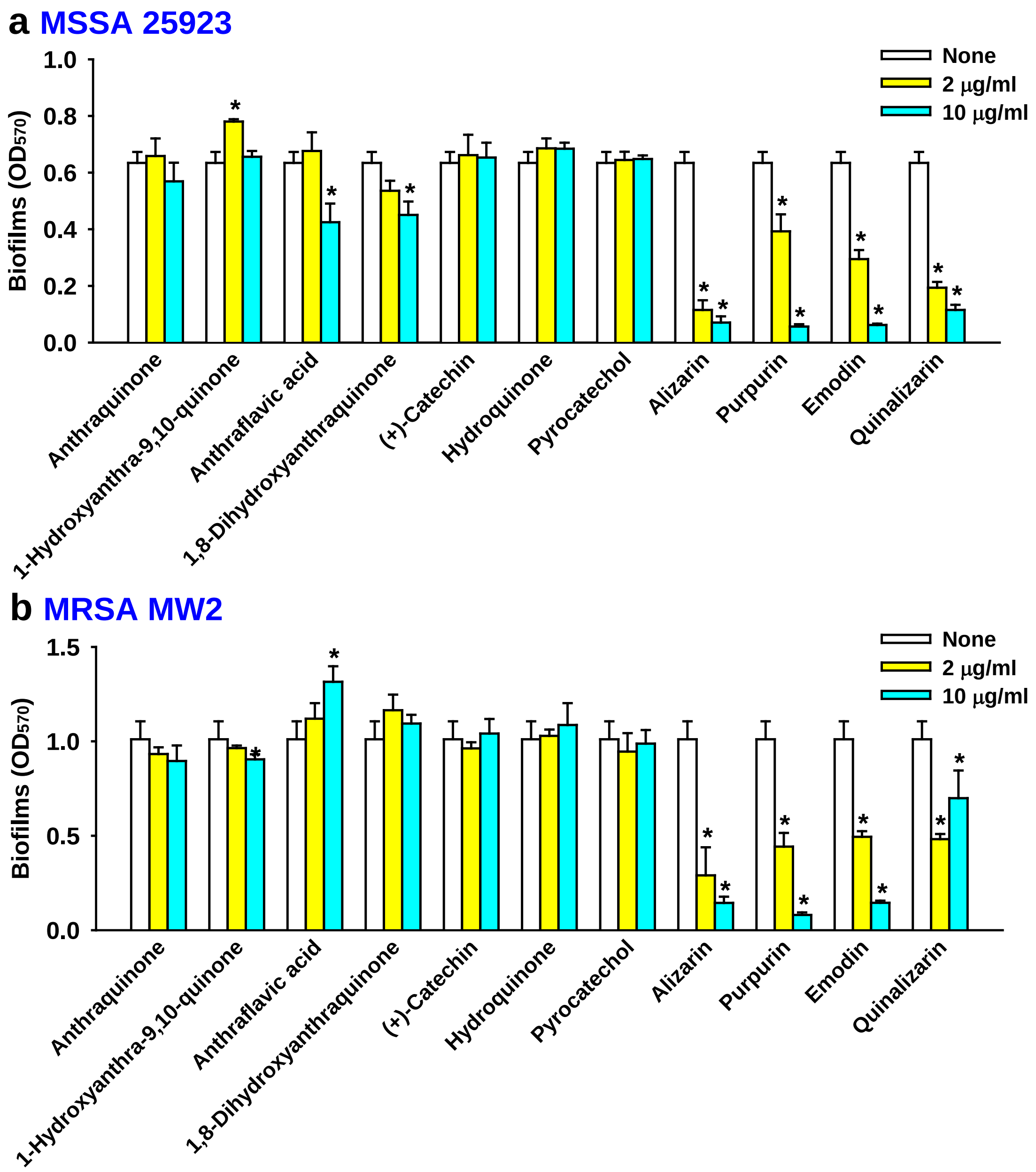

Supplementary Table S1. COMSTAT results for biofilm inhibition by alizarin against three *S. aureus* strains

Strain	Alizarin	Volume/Area ($\mu m^3\ \mu m^{-2}$)	Mean thickness (μm)	Substratum coverage (%)
MSSA 6538	None	12 ± 1	11 ± 1	99 ± 1
	2 μg/ml	7.3 ± 0.9	6.7 ± 0.7	84 ± 9
	10 μg/ml	0.9 ± 0.2	0.7 ± 0.1	32 ± 9
MSSA 25923	None	4.0 ± 0.5	3.8 ± 0.6	88 ± 10
	2 μg/ml	2.5 ± 0.3	2.7 ± 0.3	66 ± 4
	10 μg/ml	0.8 ± 0.3	0.9 ± 0.3	24 ± 9
MRSA MW2	None	5.5 ± 0.6	5.8 ± 0.9	91 ± 7
	2 μg/ml	1.7 ± 0.3	2.8 ± 0.5	35 ± 5
	10 μg/ml	1.6 ± 0.2	1.9 ± 0.2	37 ± 7

Supplementary Table S2. Cell counting in the biofilms affected by alizarin. Biofilms were produced in 96-well polystyrene plates over 24 h with or without alizarin (0, 2, 10, or 100 µg/ml) and then washed three times with PBS. Biofilms were then resuspended in 300 µl PBS, disrupted by pipetting vigorously for 60 sec and vortexing for 30 sec, and CFUs were measured. Three ind ependent experiments were conducted.

Strains	None	Alizarin 2 µg/ml	Alizarin 10 µg/ml	Alizarin 100 µg/ml
MSSA 6538	159 ± 10 x 10^6	86 ± 13 x 10^6	21 ± 2 x 10^6	N/A
MSSA 25923	167 ± 11 x 10^6	102 ± 15 x 10^6	49 ± 5 x 10^6	N/A
MRSA MW2	41 ± 6 x 10^6	10 ± 4 x 10^6	4 ± 1 x 10^6	N/A
S. epidermidis	40 ± 6 x 10^6	N/A	12 ± 2 x 10^6	5 ± 1 x 10^6

Supplementary Table S3. Sequences of the primers used for quantitative RT-PCR.

Gene	Name	Primer
16S rRNA	A component of ribosomes	Forward 5'-TGT TTG ACG ATG TTT GAG CA-3'
		Reverse 5'-CCT TCC TCC AGT TCA GAT GC -3'
agrA	Quorum-sensing regulator A	Forward 5'-TGA TAA TCC TTA TGA GGT GCT T-3'
		Reverse 5'-CAC TGT GAC TCG TAA CGA AAA-3'
aur	Zinc metalloproteinase aureolysin	Forward 5'-ACC GTG TGT TAA TTC GTG TGC TA-3'
		Reverse 5'-ATG GTC GCA CAT TCA CAA GTT T-3'
cidA	Holin-like protein	Forward 5'-AGC GTA ATT TCG GAA GCA ACA TCC-3'
		Reverse 5'-TAC CGC TAA CTT GGG TAG AAG ACG-3'
cidB	Holin-like protein	Forward 5'-TGT TTT TGT TGA CTG TCG TT-3'
		Reverse 5'-TCA TGT GAC ACT TCG ATA CC-3'
cidR	LysR-type regulator	Forward 5'-TGG TGC ATT CCA TCA ACA AT-3'
		Reverse 5'-ATT TTG CGA GTC GAT GCT CT-3'
clfB	Clumping factor B	Forward 5'-TGC AAG ATC AAA CTG TTC CT-3'
		Reverse 5'-TCG GTC TGT AAA TAA AGG TA-3'
clp9	Serine protease	Forward 5'-CAG GTA CCA TCA CTT CAT C-3'
		Reverse 5'-GGT TCA CAA ATT GAT GAC AAC G-3'
coa	Coagulase	Forward 5'-CAC GGA AAT GGC CAA GTA TC-3'
		Reverse 5'-TCG GAC GAG CTC CAT ATG AT-3'
hla	α-Hemolysin	Forward 5'-CGG CAC ATT TGC ACC AAT AAG GC-3'
		Reverse 5'-GGT TTA GCC TGG CCT TCA GC-3'
icaA	Intercellular adhesion A	Forward 5’-TGA ACC GCT TGC CAT GTG-3’
		Reverse 5’-CAC GCG TTG CTT CCA AAG A-3’
icaD	Intercellular adhesion D	Forward 5'-ACC CAA CGC TAA AAT CAT CG-3'
		Reverse 5'-GCG AAA ATG CCC ATA GTT TC-3'
icaR	Intercellular locus regulator	Forward 5'-TCG AAC TAT TCA ATT GAT GCT TTA-3'
		Reverse 5'-CAG AAA ATT CCT CAG GCG TA-3'
isaA	Transglycosylase	Forward 5'-GCT CAA ATC ATG GCT CAA CGT-3'
		Reverse 5'-TTG ATT CAC GAG CGA TGA TTG-3'
lrgA	Anti-hollin-like	Forward 5'-TGA AAC AAC AAA AAG ACG CAT CAA AAC CAG-3'

	protein	Reverse 5'-ACT TCG CCT AAC TTA ACA GCA CCA G-3'
lrgB	Anti-hollin-like protein	Forward 5'-TCG GAG GTA TTG GTA TCG-3'
		Reverse 5'-CTG CTT GAG GTA ACA TTG A-3'
nuc1	Nuclease	Forward 5'-CAC CTG AAA CAA AGC ATC CTA A-3'
		Reverse 5'-TAT ACG CTA AGC CAC GTC CAT-3'
nuc2	Nuclease	Forward 5'-ATG GAC GTG GCT TAG CGT AT-3'
		Reverse 5'-TGA CCT GAA TCA GCG TTG TC-3'
psmα	Phenol soluble modulins α	Forward 5'-ACC CAT GTG AAA GAC CTC CTT TGT-3'
		Reverse 5'-ATG GGT ATC ATC GCT GGC ATC-3'
rbf	Regulator of biofilm formation	Forward 5'-TTA GAA GGA ATC TTT AAA ACC TTA TTG AAT AA-3'
		Reverse 5'-TTG TGA ATT TTT CTT CTT CGG ACA-3'
sarA	Transcriptional regulator	Forward 5'-GAG TTG TTA TCA ATG GTC-3'
		Reverse 5'-GTT TGC TTC AGT GAT TCG-3'
sigB	RNA Polymerase sigma factor	Forward 5'-AAG TGA TTC GTA AGG ACG TCT-3'
		Reverse 5'-TCG ATA ACT ATA ACC AAA GCC T-3'
spa	Protein A	Forward 5'-ACC AGA AAC TGG TGA AGA AAA TCC-3'
		Reverse 5'-TAA CGC TGC ACC TAA GGC TAA TG-3'
